# Predictive value of serum albumin levels on cancer survival: a prospective cohort study

**DOI:** 10.3389/fonc.2024.1323192

**Published:** 2024-03-04

**Authors:** Quan Tang, Xu Li, Chun-Rong Sun

**Affiliations:** ^1^ Department of General Surgery, The Affiliated Suzhou Hospital of Nanjing Medical University, Suzhou Municipal Hospital, Gusu School, Nanjing Medical University, Suzhou, China; ^2^ Division of Cardiology, The First Affiliated Hospital of Soochow University, Suzhou, China

**Keywords:** albumin levels, cancer mortality, National Health and Nutrition Examination Survey (NHANES), cardiovascular disease (CVD), restricted cubic spline (RCS)

## Abstract

**Background:**

Serum albumin levels and cancer mortality are closely related, yet large-sample studies encompassing a broad spectrum of cancer types are lacking.

**Methods:**

This study encompassed patients diagnosed with cancer across the continuous 10 cycles of NHANES surveys from 1999 to 2018. The study population was stratified into two groups based on median albumin levels (≤ 4.2g/dL and > 4.2 g/dL) or cancer aggressiveness (well-survived cancers and poorly-survived cancers). Survival rates were estimated using the Kaplan-Meier method. The Cox proportional hazards model was employed to evaluate the association between serum albumin levels and cancer mortality. Restricted cubic spline (RCS) analysis was conducted to assess the nonlinear relationship between serum albumin levels and the risk of cancer mortality.

**Results:**

Kaplan-Meier curves demonstrated that patients with albumin levels ≤ 4.2 g/dL exhibited lower survival rates compared to those with levels > 4.2 g/dL, irrespective of cancer aggressiveness. Following adjustment for confounders, decreased albumin levels were associated with an elevated risk of cancer mortality across all groups [all cancers, HR (95%CI) = 2.03(1.73, 2.37); well survived cancers, HR (95%CI) = 1.78(1.38, 2.32); and poorly survived cancers, HR (95%CI) = 1.99(1.64, 2.42)]. RCS analyses revealed a stable nonlinear negative association between albumin levels and cancer mortality in all groups, regardless of confounder adjustment.

**Conclusion:**

Low serum albumin levels predict higher cancer mortality. Furthermore, a nonlinear negative association was observed between serum albumin levels and the risk of cancer mortality.

## Introduction

Albumin, the most prevalent protein in the bloodstream, plays a pivotal role in upholding the oncotic pressure of the blood, as well as in the binding and transportation of diverse substances and drugs (1). Additionally, it possesses anti-inflammatory, antioxidant, and antithrombotic attributes (2–5). A multitude of conditions can lead to a reduction in serum albumin levels, including malnutrition, liver injury, renal impairment, elevated catabolism, intestinal fluid loss, and more. Serum albumin levels are commonly employed as a criterion for evaluating patient nutrition status, a parameter intrinsically linked to disease outcome (6). Low serum albumin levels serve as a prognostic indicator for various diseases, including cardiovascular conditions, renal disorders, trauma cirrhosis, and COVID-19 (7–10).

Empirical evidence corroborates the significance of serum albumin levels as a potent prognostic indicator of cancer-related mortality across various patient cohorts and general populations. The 5-year survival rate for patients with Cardia adenocarcinoma, who had normal albumin levels, was significantly higher compared to those with abnormal albumin levels (38.4% vs 19.1%, P = 0.0003) (11). Colorectal cancer patients with albumin levels above 3.5 g/dL had a significantly higher 5-year survival rate compared to those with levels below 3.5 g/dL (66% vs 34%, P < 0.001) (12). Research into cause-specific mortality within the general community population has shown that low serum albumin levels are linked to increased cancer mortality (13–15). E D’Erasmo and colleagues found that serum albumin levels at admission could predict mortality and clinical outcomes at discharge in elderly patients (16). In a study involving colorectal cancer patients, an increase of 0.1 g/dL in albumin levels was associated with a 7.3% decrease in morbidity and a 15.6% decrease in mortality (17). However, these studies focusing on specific cancer types have limitations, including small sample sizes and a lack of investigation into nonlinear relationships. In this study, we utilized the NHANES (1999-2018) database to enroll a total of 4430 cancer patients in order to analyze the relationship between serum albumin levels and cancer mortality.

## Methods

### Study population

Data for this study population were obtained from the National Health and Nutrition Examination Survey (NHANES). NHANES, a platform open to the public, used a sophisticated probability sampling design to represent the non-institutionalized civilian population of the United States and was designed to assess the health and nutritional status of the U.S. population. NHANES investigated and collected a wide range of information related to demographics, lifestyle habits, diet, disease, and survival follow-up. The survey protocol was approved by the National Institute of Health Research Ethics Review Board, and all participants signed and provided informed consent. This study included patients who were diagnosed with cancer in the continuous 10 cycles of NHANES survey of 1999-2018. The exclusion criteria were as follows: i) age < 18; ii) non-cancer caused death; and iii) missing data on cancer. The study population was evenly divided into 2 groups based on median albumin (≤ 4.2 and > 4.2 g/dL). In addition, the study population was divided into two groups for comparative analysis based on the aggressiveness of the cancer [well survived cancers (Thyroid, Breast, Prostate and Non-melanoma Skin) and poorly survived cancers (Other cancer types)].

### Definition and measurement of variables

The primary outcome was cancer mortality. Survival follow-up began on the day of the participant’s interview and ended on December 31, 2019, with a median follow-up time of 85 months. All patients were diagnosed with cancer before albumin was measured. Patients’ blood samples were collected in the morning after an 8-hour fast, and then serum albumin levels were measured using the DcX800 method. Variables included age, sex, ethnicity, BMI, smoking, drinking, hypertension, diabetes mellitus, anemia, CVD were obtained by physical measurements, questionnaires, and laboratory tests. BMI was evaluated by body mass (kilograms) and body height (m^2^). The definitions of smoking and drinking referred to the Ministry of Health NZ website (18). The diagnosis of diabetes referred to the most recent ADA criteria. Hypertension was defined as self-reported by asking the question, “Have you been told by a doctor that you have hypertension?”. The diagnosis of anemia referred to the WHO criteria of men <13g/dL and women <12g/dL. The definition of CVD referred to *International Classification of Diseases* (ICD). Details on the methods of all variables are publicly available on the NHANES website (19).

### Statistical analysis

For continuous and categorical variables, baseline data were presented as the mean ± SD and percentages, respectively. The Cochran-Armitage test was used to test for between-group trends. The Kaplan-Meier method was used to estimate survival rates, and log-rank test was used to test whether the survival difference between groups was statistically significant. Univariate and multivariate Cox proportional hazards model were used to assess the association between serum albumin levels and cancer mortality. Restricted cubic spline (RCS)-fitted piecewise logistic model was used to assess the nonlinear association between serum albumin levels and the risk of cancer mortality (hazard ratio, HR). Sensitivity analysis and subgroup analysis were performed to test whether the association between serum albumin levels and cancer mortality was consistent in different subgroups or specific cancer groups. Multiple imputation (Chained equations, 25 times) was used to fill in the missing values. Full analysis was performed using Stata 17 (Stata Corp, TX, US). All tests were two-sided. Statistical significance was considered when *P* < 0.05.

## Results

### Baseline characteristics

**Figure 1:**
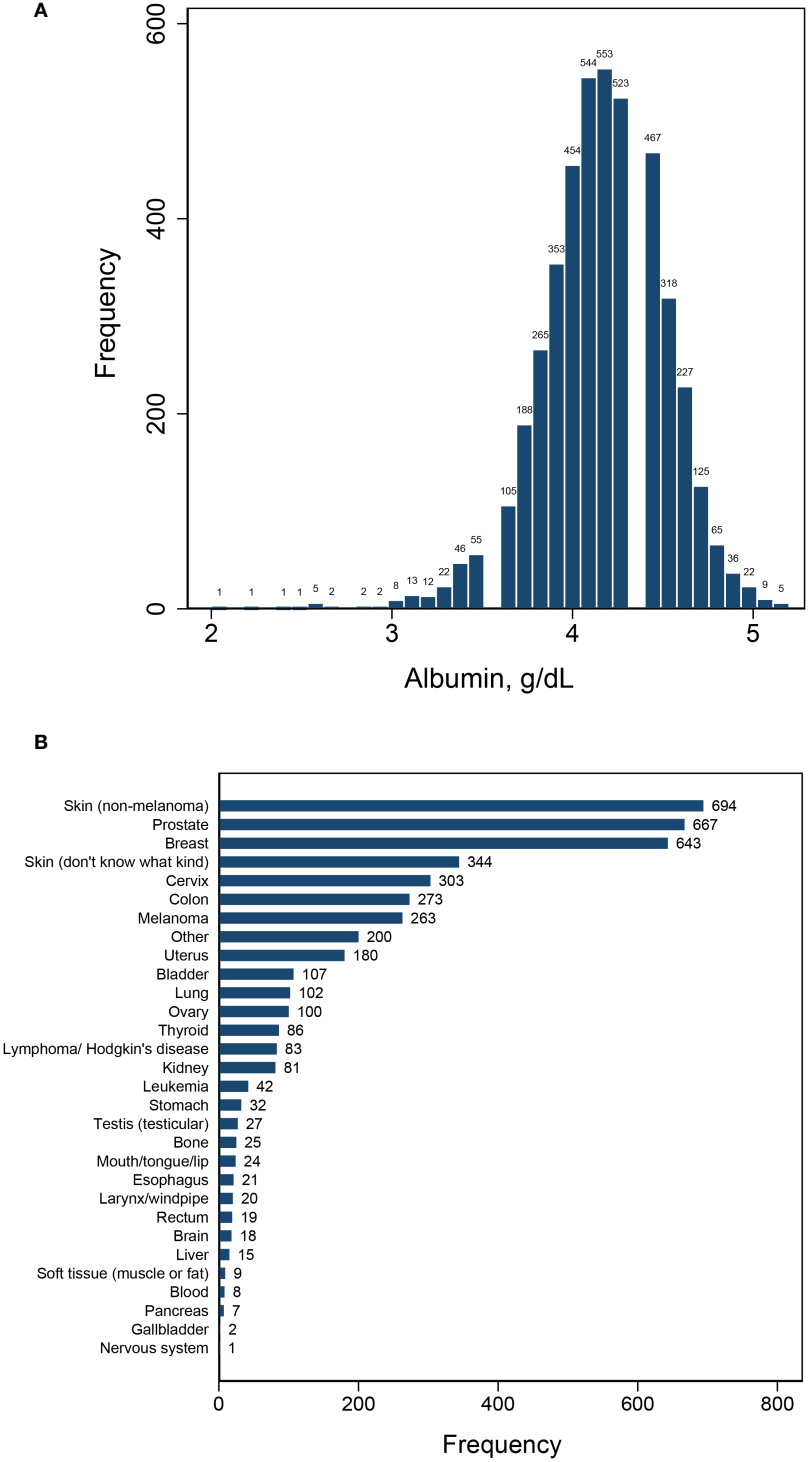
Distribution of albumin **(A)** and cancer types **(B)**.

This study encompassed a total of 4,430 patients with cancer, with 1,560 deaths occurring throughout a median follow-up period of 85 months. The patient population consisted of 2,202 individuals who survived less than 85 months and 2,228 who survived beyond that timeframe. Serum albumin levels were found to be normally distributed, with a mean ± SD of 4.16 ± 0.34 and a median (IQR) of 4.2 (0.4). A diverse range of cancer diagnoses were made, encompassing over 30 different types, led by skin cancer (non-melanoma), breast cancer, and prostate cancer (**Figure 1**). Upon segmentation based the median albumin level, there were 2,633 cases with albumin levels ≤ 4.2 g/dL and 1,797 cases with albumin levels > 4.2 g/dL (**Table 1**). Notably, cancer patients with albumin levels ≤ 4.2 g/dL were characterized by older age, a higher likelihood of being female, a greater propensity for being overweight, and a statistically significant increase in the prevalence of diabetes mellitus, hypertension, cardiovascular disease, and anemia. Furthermore, these patients exhibited a higher mortality rate compared to those with albumin levels > 4.2 g/dL (**Table 1**).

**Table 1:** Baseline characteristics stratified by albumin dichotomies.

Factor	≤ 4.20g/dL (N=2633)	> 4.20g/dL (N=1797)	P-value
Albumin, g/dL	3.95 ± 0.25	4.47 ± 0.17	<0.001
Age, year	66.91 ± 14.33	64.08 ± 14.69	<0.001
Female, sex	1507 (57.2%)	824 (45.9%)	<0.001
Ethnicity			<0.001
Mexican American	192 (7.3%)	126 (7.0%)	
Non-Hispanic White	1785 (67.8%)	1341 (74.6%)	
Non-Hispanic Black	413 (15.7%)	166 (9.2%)	
Others Hispanic	129 (4.9%)	89 (5.0%)	
Other Race	114 (4.3%)	75 (4.2%)	
BMI, kg/m²	29.72 ± 6.95	27.48 ± 5.32	<0.001
Drinking			0.270
Never	1026 (39.0%)	672 (37.4%)	
Former	951 (36.1%)	692 (38.5%)	
Current	656 (24.9%)	433 (24.1%)	
Smoking			0.450
Never	1176 (44.7%)	791 (44.0%)	
Former	1063 (40.3%)	712 (39.6%)	
Current	394 (15.0%)	294 (16.4%)	
Hypertension	1534 (58.3%)	937 (52.1%)	<0.001
Diabetes mellitus	596 (22.6%)	272 (15.1%)	<0.001
Anemia	453 (17.2%)	137 (7.6%)	<0.001
CVD	592 (22.5%)	299 (16.6%)	<0.001
Deaths	997 (37.9%)	563 (31.3%)	<0.001

Continuous and categorical variables were presented as mean ± SD or percentages, respectively. BMI, body mass index and CVD, cardiovascular disease.

### Association between serum albumin levels and cancer mortality

**Figure 2:**
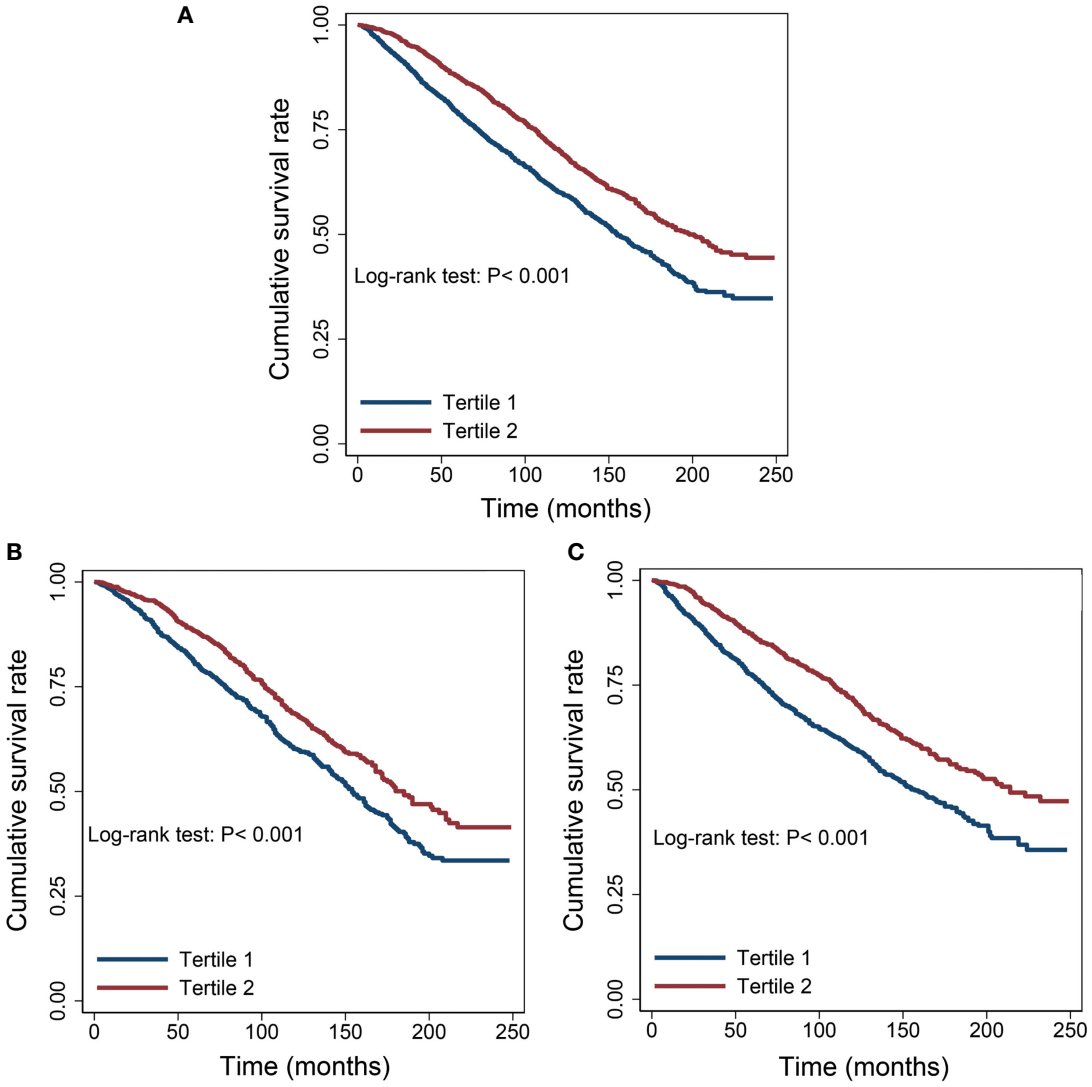
Survival differences between the low albumin group and the high albumin group. **(A)** All cancers. **(B)** Well survived cancers (Thyroid, Breast, Prostate and Non-melanoma Skin). **(C)** Poorly survived cancers (Other cancers except “**B**”).

The Kaplan-Meier survival curves revealed that patients with serum albumin levels ≤ 4.2 g/dL experienced a lower overall survival rate compared to those with levels > 4.2 g/dL, regardless of the extent of cancer malignancy (**Figure 2**).

When adjusting for potential confounders, on a continuous scale, each decrease in albumin level was associated with an elevated risk of cancer-related mortality across all patient groups [all cancers, HR (95%CI) = 2.03(1.73, 2.37); well survived cancers, HR (95%CI) = 1.78(1.38, 2.32); and poorly survived cancers, HR (95%CI) = 1.99(1.64, 2.42)] (**Table 2**).

**Table 2:** Association between serum albumin levels and cancer mortality.

Groups	Cancer mortality (Per 1g/dL albumin decrease)			
	Crude HR (95% CI)	*P* for trend	Adjusted HR (95% CI)	*P* for trend
All cancers	2.38 (2.06, 2.74)	< 0.001	2.03 (1.73, 2.37)	< 0.001
Well survived cancers	2.41 (1.89, 3.05)	< 0.001	1.78 (1.38, 2.32)	< 0.001
Poorly survived cancers	2.38 (1.99, 2.84)	< 0.001	1.99 (1.64, 2.42)	< 0.001

Crude model: adjusted for none. Adjusted model: adjusted for age, sex, ethnicity, BMI, smoking, drinking, hypertension, diabetes mellitus, anemia, CVD. HR, Hazard ratio and CI, confidence interval.

In terms of categorical analysis, multivariate Cox proportional hazards regression demonstrated that patients with albumin levels ≤ 4.2 g/dL carried a greater mortality risk than those with levels > 4.2 g/dL across all cancer groups. [all cancers, HR (95%CI) = 1.23(1.10, 1.37); well survived cancers, HR (95%CI) = 1.12(1.05, 1.30); and poorly survived cancers, HR (95%CI) = 1.30(1.12, 1.50)] (**Table 3**).

**Table 3:** Association between serum albumin levels and cancer mortality.

Groups	Albumin, g/dL	Cancer mortality			
		Crude HR (95% CI)	*P* for trend	Adjusted HR (95% CI)	*P* for trend
All cancers	≤4.20	1.46 (1.32, 1.62)	< 0.001	1.23 (1.10, 1.37)	< 0.001
	> 4.20	Reference		Reference	
Well survived cancers	≤4.20	1.37 (1.19, 1.60)	< 0.001	1.12 (1.05, 1.30)	< 0.001
	> 4.20	Reference		Reference	
Poorly survived cancers	≤4.20	1.54 (1.33, 1.78)	< 0.001	1.30 (1.12, 1.50)	< 0.001
	> 4.20	Reference		Reference	

Crude model: adjusted for none. Adjusted model: adjusted for age, sex, ethnicity, BMI, smoking, drinking, hypertension, diabetes mellitus, anemia, CVD. HR, Hazard ratio and CI, confidence interval.

**Figure 3:**
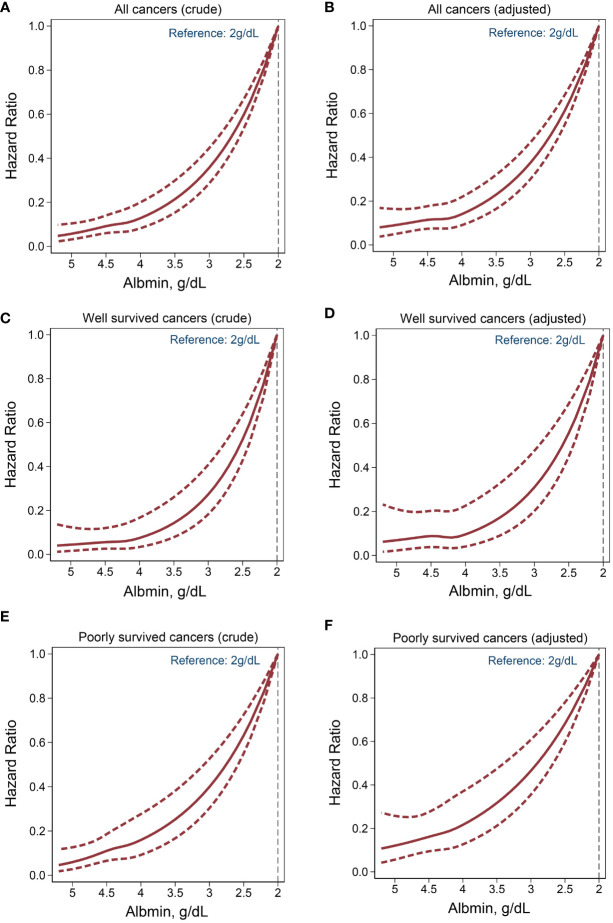
Nonlinear association between serum albumin levels and cancer mortality. Crude model: adjusted for none. Adjusted model: adjusted for age, sex, ethnicity, BMI, smoking, drinking, hypertension, diabetes mellitus, anemia, CVD. **(A, B)** All cancers. **(C, D)** Well survived cancers. **(E, F)** Poorly survived cancers.

RCS analyses highlighted a consistent and negative nonlinear relationship between albumin levels and cancer mortality risk within all patient groups, with or without the adjustment for confounding factors (**Figure 3**).

### Sensitivity and subgroup analysis

**Figure 4:**
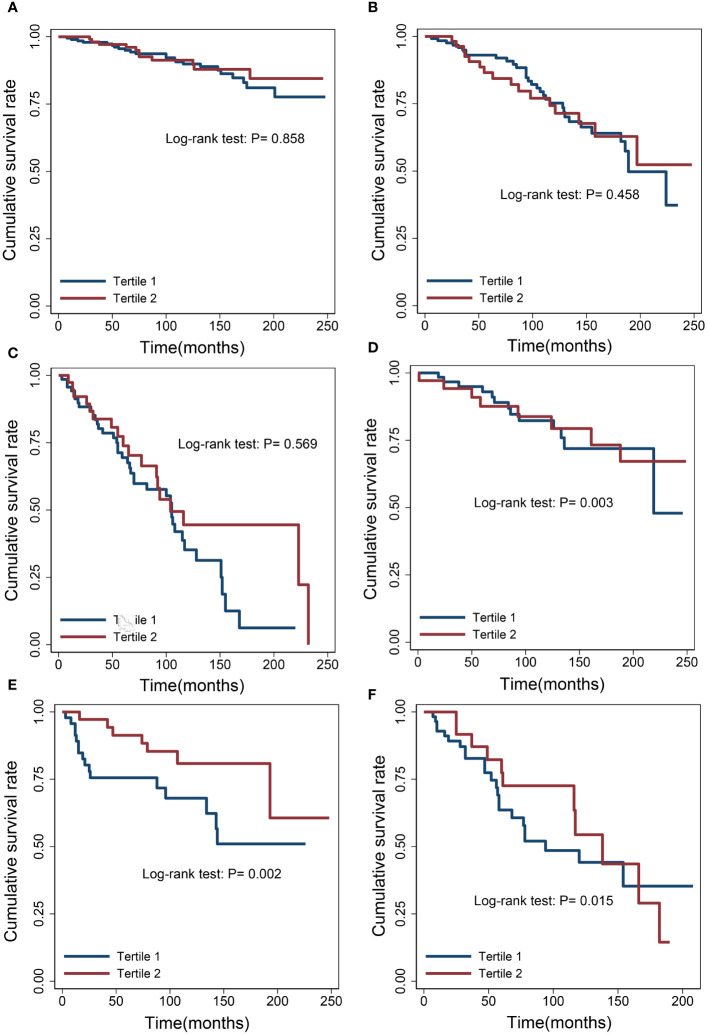
Survival differences between the low albumin group and the high albumin group. **(A)** Cervix. **(B)** Uterus. **(C)** Bladder. **(D)** Ovary. **(E)** Lymphoma. **(F)** Kidney.

We examined the relationship between albumin levels and mortality in various cancer types, including nonmelanoma skin cancer, prostate cancer, breast cancer, colorectal cancer, and melanoma, respectively, corroborating the overarching findings of this investigation (**Table 4**). Subgroup analyses further validated the associations between albumin levels and cancer mortality, as observed in the current study, across a wide range of risk factors (**Table 5**). Additionally, we conducted analyses on several rare cancer types and found no significant association between their albumin levels and cancer mortality (**Figure 4**; **Table 6**).

**Table 4:** Association between serum albumin levels and cancer mortality in specific cancer types.

Groups	N		Cancer mortality (Per 1g/dL albumin decrease)			
			Crude HR (95% CI)	*P* for trend	Adjusted HR (95% CI)	*P* for trend
Skin (non-melanoma)	694		2.97 (1.89, 4.69)	< 0.01	2.50 (1.51, 4.13)	< 0.01
Prostate	667		1.95 (1.33, 2.86)	< 0.01	1.35 (1.12, 2.04)	< 0.01
Breast	643		2.65 (1.72, 4.09)	< 0.01	1.96 (1.22, 3.08)	< 0.01
Skin (others)	344		2.33 (1.29, 4.20)	< 0.01	2.40 (1.24, 4.64)	< 0.01
Colon	273		2.43 (1.53, 3.87)	< 0.01	1.80 (1.04, 3.12)	< 0.01
Melanoma	263		5.26 (3.03, 8.92)	< 0.01	2.63 (1.37, 5.07)	< 0.01
Lung	102		3.57 (1.72, 7.35)	< 0.01	3.45 (1.38, 8.69)	< 0.01

Crude model: adjusted for none. Adjusted model: adjusted for age, sex, ethnicity, BMI, smoking, drinking, hypertension, diabetes mellitus, anemia, CVD. HR, Hazard ratio and CI, confidence interval.

**Table 5:** Association between serum albumin levels and cancer mortality in different subgroups.

Groups	Cancer mortality (Per 1g/dL albumin decrease)			
	Crude HR (95% CI)	*P* for trend	Adjusted HR (95% CI)	*P* for trend
Male	3.34 (2.76, 4.06)	< 0.01	2.17 (1.75, 2.68)	< 0.01
Female	2.38 (1.88, 2.89)	< 0.01	1.81 (1.43, 2.27)	< 0.01
Age ≤ 69	2.47 (1.87, 3.25)	< 0.01	2.39 (1.76, 3.24)	< 0.01
Age>69	2.23 (1.87, 2.65)	< 0.01	1.85 (1.56, 2.23)	< 0.01
BMI ≤ 27.8	2.86 (2.37, 3.36)	< 0.01	1.77 (1.45, 2.16)	< 0.01
BMI>27.8	2.22 (1.76, 2.79)	< 0.01	2.53 (1.94, 3.29)	< 0.01
Hypertension	2.25 (1.88, 2.69)	< 0.01	1.65 (1.25, 2.18)	< 0.01
Non-hypertension	2.34 (1.83, 2.98)	< 0.01	2.24 (1.85, 2.71)	< 0.01
Diabetes mellitus	2.41 (1.83, 3.17)	< 0.01	2.42 (1.80, 3.25)	< 0.01
Non-Diabetes mellitus	2.23 (1.89, 2.64)	< 0.01	1.90 (1.58, 2.28)	< 0.01
CVD	1.96 (1.53, 2.49)	< 0.01	1.94 (1.49, 2.54)	< 0.01
Non-CVD	2.28 (1.91, 2.72)	< 0.01	2.06 (1.70, 2.50)	< 0.01

Age and BMI were grouped according to their median. Crude model: adjusted for none. Adjusted model: adjusted for age, sex, ethnicity, BMI, smoking, drinking, hypertension, diabetes mellitus, anemia, CVD. HR, Hazard ratio and CI, confidence interval.

**Table 6:** Association between serum albumin levels and cancer mortality in rare cancer types.

Groups	N	Cancer mortality (Per 1g/dL albumin decrease)			
		Crude HR (95% CI)	*P* for trend	Adjusted HR (95% CI)	*P* for trend
Cervix	303	0.95 (0.36, 2.51)	0.930	1.07 (0.26, 4.48)	0.921
Uterus	180	2.04 (0.92, 4.55)	0.143	2.16 (0.78, 6.06)	0.143
Bladder	107	1.82 (0.82, 4.04)	0.144	0.93 (0.30, 2.94)	0.903
Ovary	100	3.45 (1.50, 7.69)	0.003	3.33 (0.70, 9.64)	0.132
Lymphoma	83	5.55 (1.59, 11.07)	0.007	3.03 (0.43, 20.12)	0.273
Kidney	81	2.17 (0.72, 6.36)	0.170	3.63 (0.78, 14.28)	0.104

Crude model: adjusted for none. Adjusted model: adjusted for age, sex, ethnicity, BMI, smoking, drinking, hypertension, diabetes mellitus, anemia, CVD. HR, Hazard ratio and CI, confidence interval.

## Discussion

This study is the first to explore the relationship between serum albumin levels and cancer-related mortality across a diverse array of cancer types in a substantial sample size. Key insights from the current investigation include: i) Reduced serum albumin levels are linked to an increased risk of cancer mortality. When accounting for confounding factors, each 1 g/dL drop in albumin levels among patients with favorable survival was associated with a 1.78-fold elevated risk, while among patients with poor survival, the risk increased by 1.99-fold; ii) A consistent and negative nonlinear association between albumin levels and cancer mortality was observed, irrespective of cancer malignancy.

Serum albumin level serves as a critical indicator of nutritional status in cancer patients and is deeply intertwined with their cancer prognosis. Previous research has consistently identified an association between albumin levels and the risk of mortality across various cancer types. For instance, Chen-Yi Wu and colleagues investigated the link between serum albumin levels and cancer mortality in community-dwelling older adults, revealing that albumin levels below 4.2 g/dL were significantly associated with increased cancer mortality compared to levels at or above 4. g/dL (15). Shuai-Shuai Xu et al. suggested that low albumin levels could be a significant risk factor for patients undergoing pancreatic cancer resection (20). Additionally, a study demonstrated that pretreatment albumin levels predicted survival outcomes in head and neck squamous cell carcinoma (17). Tilman Kühn and colleagues found that lower serum albumin levels were significantly associated with increased mortality from breast, prostate, colorectal, and lung cancer (21). A retrospective study also indicated that lower serum albumin levels predicted a higher 60-day mortality rate from acute myeloid leukemia (22). Ali Ayhan et al. identified that preoperative albumin level was an independent prognostic factor for epithelial ovarian cancer patients following optimal debulking (23). Furthermore, early postoperative serum albumin level has been shown to predict survival following nephrectomy for curative renal cancer (24). These studies corroborate the relationship between serum albumin levels and cancer mortality in cancer types, aligning with the trends observed in the current investigation.

Serum albumin is a pivotal component in certain cancer scoring systems, serving as a stabilizing factor in cancer prognosis. The Fibrinogen-to-Albumin Ratio has emerged as a potential risk predictor for patients with bladder cancer, oral cancer, gastrointestinal mesenchymal tumors, gastric cancer undergoing chemotherapy, IB-IIA cervical cancer, hepatocellular carcinoma, gallbladder cancer, and pancreatic cancer (25–32). The C-reactive Protein to Albumin Ratio has been demonstrated to predict unfavorable outcomes in colorectal cancer, oral squamous cell carcinoma, gallbladder cancer, lung cancer, and thoracic esophageal cancer (33–37). A study has highlighted the prognostic significance of the Blood Urea Nitrogen -to-Albumin Ratio in lung cancer patients receiving intensive care (38). The Albumin-Bilirubin Grade has gained importance as a tool for evaluating liver function and prognosis in patients with hepatocellular carcinoma (39, 40).

Several basic science studies have elucidated why lower albumin levels can exacerbate the risk of cancer mortality. The primary reasons include: i) The essential physiological functions of albumin; and ii) Its critical role in the context of other diseases.

Serum albumin is responsible for 80% of plasma oncotic pressure and is also the main protein in the interstitium, helping to maintain interstitial colloid osmotic pressure (41, 42). The decreased oncotic pressure due to serum albumin loss can trigger or exacerbate interstitial fluid accumulation (ascites, pleural effusion, etc.), which affects circulatory function, catabolism and anabolism, substance and drug transport, and increases the chance of infection (43–45). Therefore, oncotic pressure is a reliable predictor for measuring survival, especially in critically ill patients. The unique structure of serum albumin allows it to bind and transport various molecules, including metabolites (e.g., cholesterol, fatty acids and ions), gases (e.g., NO), and exogenous substances (e.g., drugs and dietary-derived compounds). It is well known that hypercholesterolemia increases the risk of cardiovascular disease as well as predicts their poor prognosis. In recent years, a positive correlation has also been found between serum cholesterol levels and the risk and extent of cancer development (46). Recently, targeting cholesterol metabolism for cancer treatment has been proposed (47). Animal studies showed that the release of unsaturated fatty acids is pro-inflammatory (48). Reducing unsaturated fatty acids in mouse serum can prevent lung and kidney injury, systemic inflammation, and reduces mortality (49). Thus, albumin may improve cancer survival by lowering free cholesterol and fatty acids in the blood through its binding effect. Serum albumin is also a short-term storehouse of free NO. In the presence of tissue hypoxia, albumin releases NO to maintain vascular tone (50), which may be beneficial for cancer prognosis. In addition, albumin can be used as a carrier to transport cancer-targeting drugs and food components. Decreased albumin levels directly affect the outcome and prognosis of cancer treatment. Currently, the role of recombinant albumin and albumin nanocarriers in drug delivery and cancer therapy is being extensively studied (51, 52).

Another crucial aspect of albumin’s function is its antioxidant activity in plasma. The reduced sulfur group of albumin is a major scavenger of reactive oxygen species (ROS) (53) and, in addition, albumin can limit the production of ROS by binding free copper (54). ROS have been shown to activate pro-tumor signaling in various tumors, promote cancer cell survival and proliferation, and lead to DNA damage and genetic instability (55). ROS can also modulate the tumor microenvironment, affecting various stromal cells that provide metabolic and immune support to the tumor (56). These evidences suggest that albumin may inhibit tumor growth and metastasis through antioxidant pathways, thereby improving prognosis.

Serum albumin can also interact with other pathways to exert anti-inflammatory effects (5). Studies have demonstrated that serum albumin levels predict the prognosis of acute inflammatory conditions such as primary trauma, burns, or acute infections (57–59). In addition, studies have found that albumin has anticoagulant and antiplatelet properties, but evidence for this is scarce (3, 4). Albumin may improve the long-term survival of cancer patients by reducing disease complications and unexpected deaths through anti-inflammatory and anti-thrombotic effects.

Serum albumin is a valuable biomarker for a variety of pathological conditions, including inflammation, ischemia, autoimmunity, and metabolic disorders (42). As already mentioned above, lower albumin levels lead to a decrease in the body’s anti-inflammatory, antioxidant, and antithrombotic capacity, which further contributes to the poor prognosis of the disease. Pre-existing hypoproteinemia is a commonly used prognostic indicator indicating a worse prognosis for a wide range of diseases from medical conditions to surgery (60, 61). A very large body of evidence supports the prognostic role of serum albumin levels in cardiovascular disease (62, 63), abdominal surgical disease (64, 65), orthopedic disease (66, 67), gynecological disease (68, 69), and infectious disease (70). Therefore, reduced albumin levels may indirectly affect the prognosis of cancer by promoting the development of other diseases.

This study uncovers a nonlinear relationship between serum albumin levels and cancer mortality risk, suggesting that the risk of mortality triggered by a decrease in albumin levels is ampl at lower levels of albumin, thus exhibiting threshold effects. Albumin infusion is primarily used for plasma replacement and rehydration in critically ill patients, as well as treating hepatic diseases such as ascites, spontaneous bacterial peritonitis, hepatorenal syndrome, and post-perforation syndrome (71). However, many conditions with reduced albumin levels are considered unsuitable for albumin infusion due to the lack of significant benefits in terms of prognosis and reduced mortality in several previous basic studies and clinical trials (72–76). The threshold effect identified in this study may help explain the ineffectiveness of albumin infusion under certain circumstances.

This study demonstrates that albumin is a significant predictor of cancer mortality in both aggressive and less aggressive cancers, supporting its potential as a stable risk predictor for the most common cancers. Furthermore, the nonlinear relationship suggests the importance of choosing the appropriate timing for albumin infusion in cancer patients. In terms of clinical research, future studies should refine the inclusion criteria, expand the sample size, focus on exploring the relationship between albumin and the prognosis of specific cancer types, particularly the nonlinear relationship. Moreover, efforts should be made to fully develop and utilize various scoring models related to albumin to better serve cancer patients. In terms of basic research, future studies should further investigate the links between albumin and the pathological mechanisms of cancer to better understand its role in cancer prognosis.

### Limitations

Firstly, the primary findings of this study focus on the relationship between albumin levels and overall cancer mortality, which can be applied to various aggressive and less aggressive cancers, but not to specific cancer types. Although we examined some specific cancer types, the limited sample size may result in findings that need to be further validated. Secondly, inconsistencies in lifestyle and indicator measurements among patients in different cycles may lead to measurement bias. Some covariates selected for this study, such as smoking, alcohol consumption, hypertension, and diabetes mellitus, were obtained through subjective questionnaires, which may have resulted in unavoidable information recall bias. Lastly, due to the limitations of the database, several confounding factors that may influence cancer mortality, such as treatment modality, drug use, and compliance, were not adjusted for.

## Conclusions

Low serum albumin levels predict higher cancer mortality. Furthermore, a nonlinear negative association was observed between serum albumin levels and the risk of cancer mortality.

## Data availability statement

The original contributions presented in the study are included in the article/supplementary material. Further inquiries can be directed to the corresponding author.

## Ethics statement

The studies involving humans were approved by the National Institute of Health Research Ethics Review Board. The studies were conducted in accordance with the local legislation and institutional requirements. The participants provided their written informed consent to participate in this study.

## Author contributions

QT: Writing – original draft, Writing – review & editing. XL: Writing – original draft, Writing – review & editing. CS: Project administration, Supervision, Writing – original draft, Writing – review & editing.
